# Functional and structural brain modifications induced by oculomotor training in patients with age-related macular degeneration

**DOI:** 10.3389/fpsyg.2013.00428

**Published:** 2013-07-17

**Authors:** Katharina Rosengarth, Ingo Keck, Sabine Brandl-Rühle, Jozef Frolo, Karsten Hufendiek, Mark W. Greenlee, Tina Plank

**Affiliations:** ^1^Institute for Experimental Psychology, University of RegensburgRegensburg, Germany; ^2^Department of Ophthalmology, University Medical Center RegensburgRegensburg, Germany

**Keywords:** age-related macular degeneration, fMRI BOLD, voxel-based morphometry, cortical plasticity, aging

## Abstract

Patients with age-related macular degeneration (AMD) are reliant on their peripheral visual field. Oculomotor training can help them to find the best area on intact peripheral retina and to efficiently stabilize eccentric fixation. In this study, nine patients with AMD were trained over a period of 6 months using oculomotor training protocols to improve fixation stability. They were followed over an additional period of 6 months, where they completed an auditory memory training as a sham training. In this cross-over design five patients started with the sham training and four with the oculomotor training. Seven healthy age-matched subjects, who did not take part in any training procedure, served as controls. During the 6 months of training the AMD subjects and the control group took part in three functional and structural magnetic resonance imaging (MRI) sessions to assess training-related changes in the brain function and structure. The sham-training phase was accompanied by two more fMRI measurements, resulting in five MRI sessions at intervals of 3 months for all participants. Despite substantial variability in the training effects, on average, AMD patients benefited from the training measurements as indexed by significant improvements in their fixation stability, visual acuity, and reading speed. The patients showed a significant positive correlation between brain activation changes and improvements in fixation stability in the visual cortex during training. These correlations were less pronounced on the long-term after training had ceased. We also found a significant increase in gray and white matter in the posterior cerebellum after training in the patient group. Our results show that functional and structural brain changes can be associated, at least on the short-term, with benefits of oculomotor and/or reading training in patients with central scotomata resulting from AMD.

## Introduction

Age-related macular degeneration (AMD) is among the most common causes for vision loss and blindness in developed countries (Liu et al., 2012). The visual deficit in AMD is characterized by atrophy of photoreceptor cells in the patients' macula resulting in a complete foveal scotoma. As a consequence these patients are forced to develop specific coping strategies concerning their visual loss. Many patients try to compensate for their impaired central vision by using strategies of eccentric viewing to manage daily visual tasks like reading. AMD patients often develop a pseudo fovea at a specific point in their eccentric retina, the so-called “preferred retinal locus” (PRL; Bäckman and Inde, 1979; Timberlake et al., 1987; Whittaker et al., 1988; Guez et al., 1993; Fletcher and Schuchard, 1997). The establishment of a PRL could be regarded as a consequence of an intense, though mostly implicit, form of perceptual learning. It is well-known that perceptual learning can lead to a permanent change in many aspects of visual performance in subjects with intact vision (e.g., Karni and Sagi, 1991; Schoups et al., 1995; Ahissar and Hochstein, 1997; Gilbert et al., 2001; Watanabe et al., 2001; Fahle and Poggio, 2002). Age might play a role here, since older persons might be less efficient in developing a PRL compared to younger persons. In a longitudinal study, Rovner et al. (2009) pointed out that AMD patients relinquish cognitive, physical and social activities due to their visual deficit, leading to an increased risk of cognitive decline. A review study by Mitchell and Bradley (2006) described specific training interventions for AMD patients to ensure a desirable quality of life. An oculomotor training program might be a suitable approach to train AMD patients to establish the best possible area in intact peripheral retina and to efficiently stabilize eccentric fixation (e.g., Bäckman and Inde, 1979; Nilsson et al., 2003; Coco-Martín et al., 2013). Seiple et al. (2005) showed that eccentric reading training in patients with AMD could improve their fixation quality and eye movement control. Such a training-induced improvement might be reflected in corresponding changes on a functional and structural level in the brain. Two recent voxel-based morphometry (VBM) studies in patients with juvenile macular dystrophy Plank et al. (2011) and in patients with AMD (Hernowo et al., 2013) reported a significant decrease in gray matter volume in the lesion projection zone in the patients' primary visual cortex. Similar findings were demonstrated by Boucard et al. (2009) for patients with AMD or glaucoma.

In this study we investigated in 9 AMD patients with respect to the extent to which improvements in the efficient use of the PRL due to intensive oculomotor training are reflected in functional and structural neural changes. Participants completed an oculomotor training over a period of 6 months and a non-visual sham training (auditory memory training) for another 6 months in a cross-over design. Can oculomotor training improve fixation stability, reading speed and visual acuity in our patients with bilateral central scotoma resulting from AMD? If this is the case such training should also impact on the quality of these patients' everyday lives. In this study we examined how such behavioral changes correlate with functional and structural neural changes. In an fMRI study the patients' PRL and an untrained area in the opposite hemifield (OppPRL) were stimulated using either natural images of everyday objects or checkerboard stimuli. We expected to find an increase in the BOLD signal with oculomotor training effects in the PRL projection zone, but not in the projection zone of the untrained hemifield. In another fMRI study in our lab, where patients with hereditary retinal dystrophies performed a visual search task (Plank et al., 2012), we found that BOLD responses in early visual cortex were significantly up-regulated in patients with stable eccentric fixation in comparison to patients with unstable fixation. Since patients vary with respect to their success in the oculomotor training, we also expected to find a positive correlation between the change in fixation stability and the training-induced change in BOLD signal in visual cortex. Additionally anatomical scans were acquired in each MRI session to determine structural changes, where an increase in gray and white matter would reflect a positive consequence of successful fixation training.

## Methods

### Patients and control subjects

Nine patients with diagnosed AMD and seven age-matched controls participated in the study. All participants signed an informed consent form prior to the participating in the study and they received modest monetary compensation for their participation. The study was approved by the Ethical Committee of the University of Regensburg and conducted in accordance to the ethical guidelines of the Declaration of Helsinki.

### Ophthalmologic methods and examinations

**Figure 1:**
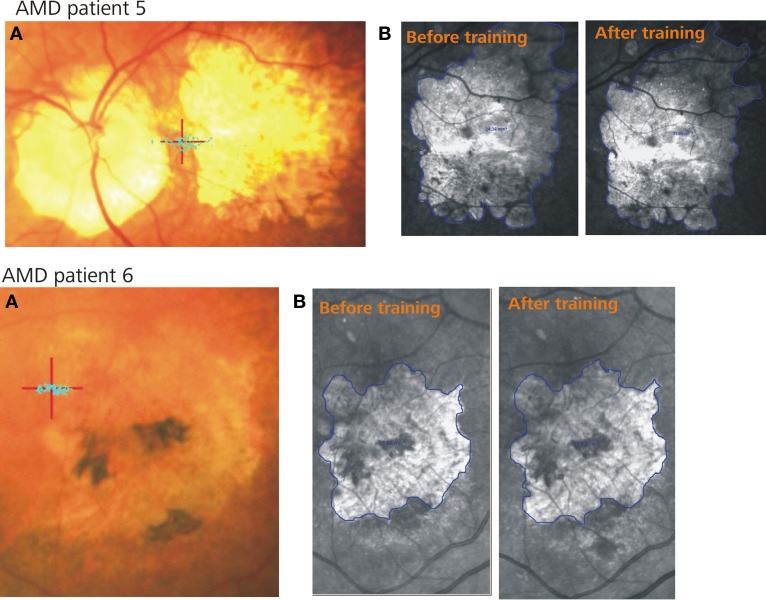
**Two example patients. (A)** Shows the PRL (red cross) and the fixation stability (blue dots) after training, **(B)** represents the infrared images of the retina including the scotoma (blue lines) before (left) and after (right) training.

The initial ophthalmologic examination included mydriatic fundus camera imaging as well as fundus autofluorescence (FAF) and infrared reflection (IR) imaging of the macular pathology (for examples see Figure 1). Before each fMRI visit, microperimetry and non-mydriatic fundus imaging were performed including either IR or, in selected cases, FAF.

Both IR and FAF imaging were conducted using the Spectralis Heidelberg Retina Angiograph 1 (Heidelberg Engineering, Heidelberg, Germany) confocal laser-scanning ophthalmoscope (cSLO).

IR imaging is a variation of fundus photography using infrared light instead of white light or spectral colors for illumination. The infrared light penetrates unclear media better than white light, which is an advantage when imaging patients with cataracts. Since reflectance increases with wavelength, near infrared wavelengths have a better fundus reflectance than wavelengths from the visible spectrum and lead to deeper light penetration into the retina. IR imaging with SLO technology provides high contrast and allows for assessment of pathologies reaching into the extreme outer layers of the retina and the choroid. FAF is a well-established technique for the detection of alterations of the retinal pigment epithelium (RPE) based on changes of lipofuscin distribution in RPE cells. In geographic atrophy, the lost tissue is marked by hypofluorescence due to the loss of fluorophores, typically surrounded by a hyperfluorescent rim with accumulation of fluorophores. This aspect facilitates precise area measurement of the retinal lesions. The progression of geographic atrophy was measured by comparing a baseline IR or FAF image with one made at last visit. Mean follow-up was 14.7 months (range 7–20 months). On IR images, the area of atrophy was selected manually before calculating the size in mm^2^ with graphical software. The size of the atrophic area on the FAF images was calculated with the Region Finder software tool.

### Clinical characteristics and visual field measurements

Table 1 presents details on demographic characteristics of patients and controls, including the gender, age, duration of disease at time of study, scotoma size, and position of PRL in the visual field.

**Table 1:** **Demographic characteristics of AMD patients (AMD 1-AMD 9) and matched controls (C 1–C 7) referring to their gender, age, duration of disease, scotoma size, study eye and location of the PRL in patients' visual field**.

**Subject**	**Gender**	**Age**	**Duration of disease (in years)**	**Scotoma size (diameter in degrees)**	**Position of PRL in visual field**	**Study eye**
AMD 1	Male	55	3	15	Left	OD
AMD 2	Male	62	5	20	Left	OS
AMD 3	Female	70	2	15	Lower	OD
AMD 4	Female	80	15	10	Left	OS
AMD 5	Male	63	4	10	Left	OS
AMD 6	Male	79	8	15	Left	OD
AMD 7	Female	84	6	15	Lower	OS
AMD 8	Female	81	21	10	Lower	OS
AMD 9	Female	71	10	10	Right	OS
C1	Female	51				OD
C2	Female	62				OS
C3	Female	72				OD
C4	Male	83				OS
C5	Female	64				OS
C6	Female	78				OD
C7	Male	71				OS

Scotoma size was measured using kinetic Goldmann perimetry with the isopters III/4e, I/4e, I/3e, I/2e, and I/1e. Defined as edges of the scotomata, those points were marked, where isopter III/4e were no longer detected. Scotoma size is reported in Table 1 as scotoma diameter in degrees of visual angle as an average of vertical and horizontal dimensions. Controls did not undergo Goldmann perimetry. Visual acuity, fixation stability, and reading speed were measured before, during and after training to indicate improvements induced by training.

Best-corrected visual acuity was determined by using a Vision Screener (Rodenstock Rodavist 524/S1) and Eye Charts for distant (Oculus Nr. 4616) and near visual acuity (Zeiss/Frohnhäuser). For the patient group the dominant eye was chosen as the study eye, for the controls the same eye as that used by their age-matched patient was chosen as the study eye.

We used a Nidek MP-1 microperimeter (Nidek Co, Japan) to measure fixation stability. Patients were requested to fixate (eccentrically) a red cross of 4 degrees visual angle in diameter for approx. 30 s, whereas controls fixated the target with their fovea. The technique measures 25 samples per second, resulting in 750 fixation samples over 30 s. During the measurement the camera sometimes lost track of the subject's eye. This can be due to eye blinks or fixation instability in the form of large saccades. The Nidek software records the time period that was measured and the proportion of the time span that was effectively tracked, as well as the percentages of fixation points that fell in a range of 2° or 4° diameter visual angle around the center of the fixation target, based on the time spans effectively tracked. Thus, fixation stability can be overestimated by long or frequent time spans where the camera had lost track of eye position due to large saccades. To compensate for this we corrected the given fixation stability in the following way (see Plank et al., 2011): first we calculated the mean time span for which the camera lost track of eye position in the normally sighted control group, who fixated with their fovea. The resulting mean value of 13 s (*SE* = 10.3 s) yielded an estimate of the time that could be attributed to eye blinks. In a second step we subtracted this amount from the measured time, in which the camera had lost track of the eye in the patient's group. The difference between the measured time remaining and the effectively tracked time we attribute to large saccades. This time span was added to the effectively tracked time. On this basis we recalculated the percentages of fixation points falling in a range of 2° visual angle around the target.

To measure reading speed patients read aloud a continuous text for 3 min, which was recorded. We then counted the number of words read and calculated the mean of words read per minute. All participants read the same text, taken from a book [German translation of Doris Lessing (2003): The Grandmothers], but different passages of the same book at each session, printed on a sheet of paper (font: Arial, font size: 10 pt, single spaced). Patients used magnification glasses customized to their needs. The same magnification was used for all reading test sessions, except for one session of patient AMD 9 (session “memory 1” conducted 3 months after the completion of the oculomotor training), who needed greater magnification in that session due to some minor disease progression.

To assess the patients' own perception of their visual function before and after eccentric viewing training we used the National Eye Institute's Visual Function Questionnaire (VFQ-25; Mangione et al., 2001) in its German translation.

### Training procedure

The training procedure was implemented as a crossover design consisting of two parts, with an average duration of 6 months each. One part was a direct training of the PRL, the other part was a pseudo-training in the form of an auditory memory training. Participating patients completed both parts, half of the patients started with the direct training of the PRL, the other half with the pseudo-training. Patients were randomly assigned to one of the two groups.

### Direct training of PRL

To train the patients to establish, uphold and stabilize eccentric fixation we used techniques described by Bäckman and Inde (1979), Nilsson and Nilsson (1986), Nilsson et al. (2003), Gustafsson and Inde (2004), Seiple et al. (2005), Kasten et al. (2010). All training was conducted with one eye only, the chosen study eye (see Table 1), while the other eye was patched. In an initial phase we used the computer program Xcentric Viewing as described by Kasten et al. (2010), applying the program parts as recommended. At first we determined a prime viewing position in the peripheral visual field that was determined to be the best position for the patient's PRL. While the patient was asked to fixate the center of the screen, marked by crosshairs, at several positions in the peripheral visual field single letters and short words were presented in different sizes. The patient gave feedback about the readability of those stimuli and thus the best position was determined for training. Three patients (AMD 1, 6, and 9) did not have distinct regions on the peripheral retina that they used for viewing in daily life prior to training, so that a new “PRL” had to be determined. One patient (AMD 5) had some preference for directing his gaze to see eccentrically, but was retrained to a slightly different retinal location since the new “PRL” provided better reading performance. Five patients (AMD 2, 3, 4, 7, and 8) of our sample had already chosen a certain area as their PRL that also turned out to be the best peripheral region to train. During training the therapist (author Sabine Brandl-Rühle) presented letters, digits, words, and running text in different sizes at the chosen PRL. The font size was decreased and the word length increased in dependence of the individual improvements of the participants. Additionally the patients were asked to train their eccentric fixation in a similar manner at home (for a time spans of 10 min several times a day), using a simplified version of the software. Adequate optical magnification was provided. When the patients were able to read long words in small font size on the computer screen, the training passed on to reading printed text. As described by others, e.g., in Bäckman and Inde (1979), Nilsson et al. (2003), Gustafsson and Inde (2004), and Kasten et al. (2010), patients were trained to use the “moving text” technique with the aid of optical lenses and fixation lines. Subsequently, the patients were trained to exert oculomotor control using eye movement tasks as described in Seiple et al. (2005). Participants practiced executing visually-guided saccades with their PRL, starting with small horizontal saccades between simple stimuli like dots, passing on to larger saccades, horizontally as well as in a clockwise manner, while viewing more complex stimuli like single letters or two- to three-letter words. The training paradigms were implemented as described in Seiple et al. (2005), using the software Presentation (Neurobehavioral Systems) as well as a simplified version, implemented in C++ for use at home. Accordingly, patients were asked to practice the tasks at home for a recommended time span of 15–20 min daily. Eye movements and fixation stability were monitored using a video eyetracker (High Speed Video Eyetracker Toolbox, Cambridge Research Systems, UK) throughout the training whenever the computer-based training protocols were in use.

Twelve regular training sessions were scheduled over a 6-month period. Training sessions were more densely timed at the beginning of the training with weekly sessions to accompany the initial stabilization process of the more efficient eccentric viewing, while they were more spread out (at intervals of 2–3 weeks) at the end of the training schedule when patients already had established their PRL. Patient AMD6 had no computer at home, therefore he trained 1 h twice a week with the therapist instead of home training. Patient AMD7 had only 6 sessions with the therapist and practiced more at home due to the long distance between her home and the clinic.

### Sham-training

As sham-training for a further period of 6 months patients completed an auditory memory training (in German, *Gedächtnistraining für blinde Senioren, Memory training for the elderly blind*; Bernard, 2000), provided on audio-CDs. Participants were asked to complete two training units a week for about 10 min each. Participants were also asked to fill in questionnaires related to the memory exercises and to repeat training units as needed.

### Training schedule and fMRI measurements

Direct training of the PRL and pseudo-training were accompanied by fMRI sessions as described above. Functional MRI measurements were repeated five times during the entire period of fixation and sham training. In a crossover design fixation training was either preceded by or followed by such sham training to control for placebo effects.

Due to vacation or critical life events, intervals between single sessions were sometimes longer, but they never exceeded 5 months. AMD1 suffered a minor stroke between session 4 and 5 (during auditory memory training phase), but recovered fully. For AMD5 only the PRL was stimulated in session 1, not the OppPRL, because it was not possible to determine the position of the PRL in relation to the fovea precisely. Therefore, an additional fMRI session was conducted 7 weeks after the first session and 7 weeks before the second session. For AMD8 the hourly fMRI sessions were split in pairs of 30-min sessions, completed on two different days, in each case not more than 2 days apart from each other, due to fatigue reported by the patient during hour-long sessions.

### Stimuli and procedure

**Figure 2:**
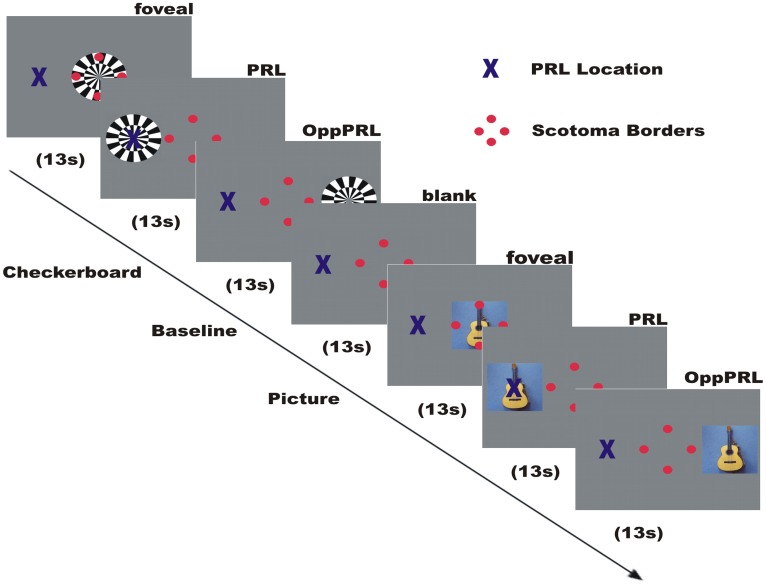
**Illustration of the time course of a typical run in an fMRI session**. Direct stimulation was conducted in the fovea, the PRL, and the opposite PRL either with checkerboard or images of everyday objects. Blue cross marks the preferred retinal locus of fixation in the AMD patient. The 4 red dots were positioned at the scotoma rim and aided stable fixation.

The functional MRI measurements consisted of retinotopic mapping paradigms and a direct stimulation of patients' PRL. Here we primarily report the results of the direct stimulation of the patients' PRL and of a comparably peripheral area in the opposite hemifield (OppPRL) with flickering checkerboards and meaningful pictures of everyday objects. Additionally meridian mapping was employed to functionally determine the locations of visual areas V1, V2, and V3 for each participant. Visual stimuli were projected onto a circular screen (31° visual angle in diameter at a distance of 60 cm) placed behind the head of the participant at the end of the scanner bore and visible via a mirror placed within the MRI head coil. All visual sequences were presented with the software Presentation (Neurobehavioral Systems Inc.) and triggered by the scanner signal upon onset of volume acquisition. As in behavioral testing, the participants conducted all paradigms monocularly with their study eye, the other eye was patched during the measurement. All participants had to fixate at the center of the screen during all tasks. Controls fixated foveally a central fixation target (the letter “X”). For patients with central visual field scotomata we presented auxiliary stimuli to ensure fixation. These auxiliary stimuli were adapted to the individual needs of the patients depending on how well they could consciously perceive their scotoma and/or how well they were accustomed to fixate with their PRL. The auxiliary stimuli consisted of four red dots (one about 0.7° visual angle in diameter) positioned at the edges of the respective scotoma and/or the fixation target (letter “X”) at the position of the PRL (see Figure 2). The fixation target was located at a position in the visual field that corresponded to the PRL and was adapted in size to the needs of the patients (between 0.95° and 2.1° visual angle). The positioning of the auxiliary stimuli on the screen and the direction of gaze was controlled prior to scanning by using a video eyetracker (MREyetracker, Cambridge Research Systems, UK) under comparable viewing conditions as during fMRI.

### Meridian mapping

In this paradigm we measured the cortical responses to the presentation of a pair of collinearly aligned wedges consisting of flickering checkerboards that were positioned at either the horizontal or vertical meridian with the goal to identify the borders of the retinotopic areas V1, V2, and V3 (DeYoe et al., 1996; Beer et al., 2009), as described in Plank et al. (2012). Black (1 cd/m^2^) and white (330 cd/m^2^) checkerboard stimuli with a flicker rate of 8 Hz were presented sequentially, covering the horizontal and vertical meridian, on a gray background of mean luminance in a block design together with a baseline condition of mean luminance. The blocks were presented over eight cycles, each cycle consisting of flickering checkerboards for 19 s and a blank baseline condition for 18 s. Auxiliary stimuli and fixation target were visible throughout the stimulation. The representations of the horizontal and vertical meridians on the visual cortex were then identified by computing the contrast between the horizontal and vertical meridian conditions. Vertical meridian representations marked the borders between V1 and V2, and horizontal meridian representations marked the borders between V2 and V3. The borders between dorsal and ventral regions within the same visual area were set at the midline of the representation of the horizontal meridian coinciding with the calcarine sulcus.

### Direct stimulation of PRL

In this paradigm we stimulated the PRL of the patients directly, as well as a comparable peripheral area in the opposite hemifield (OppPRL) and the central area in the visual field with flickering checkerboards and pictures of everyday objects. By this means we wanted to identify possible differences in the projection zones for the PRL and OppPRL in the visual cortex in response to meaningful (pictures of everyday objects) and not meaningful, abstract (checkerboards) stimuli. The participants were instructed to view the stimuli and try to recognize the objects presented in the pictures. Stimuli in this paradigm were either a disk of black and white checkerboards (size: 9° × 9° visual angle) presented with a flicker rate of 8 Hz or chromatic images of natural objects (e.g., animals, tools, vehicles, musical instruments; 7.3° × 7.3° visual angle). Overall 72 different pictures were presented in each session in a random order. The photographs were collected from free Internet databases or taken by the authors. Stimuli were presented blockwise on a gray background, together with a baseline condition (gray background of medium luminance). The blocks were presented in four repetitions, the sequence of one repetition is depicted in Figure 2. Flickering checkerboards and meaningful pictures were presented in blocks of 13 s each, the baseline condition (blank screen) in blocks of 18 s. In a block with meaningful pictures the picture changed every 2.2 s without a gap, so that six different pictures were presented sequentially in each object block. In each block one of three positions in the visual field was stimulated: center, PRL or OppPRL. The exact location of PRL was determined individually. As OppPRL we used comparable coordinates in the opposite hemifield. Auxiliary stimuli and the fixation target were visible throughout the stimulation.

### Magnetic resonance imaging

Image acquisition was conducted on a 3T head scanner (Allegra Siemens, Erlangen, Germany).

Functional images were acquired using a T2^*^-weighted gradient echo-planar imaging (EPI) sequence (*TR* = 2 s, *TE* = 30 ms, 34 slices, FoV = 192 × 192 mm^2^, flip angle = 90°, 3 × 3 × 3 mm^3^ voxel size). The axial slices were oriented parallel to the plane connecting the anterior and posterior commissure and covered the whole brain. Two dummy scans at the beginning of each measurement were removed automatically from the data set. Additionally a high-resolution T1-weighted image was acquired before training, during training and after training of fixation stability. We used a modified version of the MP-RAGE (3D magnetization prepared rapid gradient echo) sequence from “The Alzheimer's Disease Neuroimaging Initiative” (Jack et al., 2008). We obtained 160 slices with a resolution of 1 × 1 × 1 mm^3^ using a FOV = 256 × 256 mm^2^. The TR was 2250 ms, the TE 2.6 ms and the flip angle 9°. Functional T2^*^ weighted images were also acquired before, during and after training. This resulted in five scanning sessions in total: the patient group that started with the oculomotor training had one session before any training, one session during training and one after training. Only one patient (AMD 5) was measured one additional time during training. Further two sessions were completed while the patients underwent sham training (auditory memory training). The group that started with the sham training had two sessions while conducting the sham training, subsequently one session before the start of the oculomotor training, one session during oculomotor training and one session after oculomotor training.

### MRI data analysis

MRI data analysis was performed as described in Plank et al. (2012) and as is summarized below.

### Analysis of functional data

#### Cortical reconstruction

The T1-weighted structural image obtained from each subject was reconstructed by Freesurfer version 4.1 (Martinos Center for Biomedical Imaging, Charlestown, MA) as described in Beer et al. (2009, 2011). The cortical reconstruction procedure included the removal of non-brain tissue with a hybrid watershed/surface deformation procedure (Segonne et al., 2004), correction for intensity non-uniformities (Sled et al., 1998), and automatic transformation into Talairach space. After segmentation of the subcortical white matter and deep gray matter volumetric structures (Fischl et al., 2002, 2004), the gray-white matter boundary was tessellated and topologic inaccuracies automatically corrected (Fischl et al., 2001; Segonne et al., 2007). The surface was then deformed following intensity gradients to optimally place the gray/white and gray/cerebrospinal fluid borders at the location where the greatest shift in intensity defines the transition to the other tissue class (Dale et al., 1999; Fischl and Dale, 2000). Once the cortical models were complete, the cortical surface was inflated (Fischl et al., 1999a), registered to a spherical atlas which utilized individual cortical folding patterns to match cortical geometry across subjects (Fischl et al., 1999b), and automatically parcellated into units based on gyral and sulcal structures (Fischl et al., 2004; Desikan et al., 2006). Finally we created an occipital flat patch of the inflated surface posterior to the sylvian fissure (cut along the calcarine sulcus).

### Preprocessing of functional data

Data analysis was performed with the FS-Fast tools of Freesurfer. Pre-processing steps included motion correction (Cox and Jesmanowicz, 1999), co-registration to the anatomical image acquired in the same session, smoothing with a Gaussian kernel of 5 mm FWHM and correction for intensity non-uniformities (Sled et al., 1998). Additionally, the first volume of each session was automatically co-registered to the structural volume and manually verified (and corrected) using visual (“blink”) comparison.

### Statistical data analysis and ROI labeling

To obtain functional estimates of the anatomical borders between visual areas V1 and V2 as well as between V2 and V3 we contrasted conditions of horizontal meridian representations with vertical meridian stimulations (see also Beer et al., 2009). Statistical parametric maps were calculated based on the general linear model using the stimulation blocks convoluted with a cumulative gamma function (with parameters: delta = 2.25; tau = 1.25; alpha = 2) as predictors. Additionally, linear and cubic predictors (modeling slow signal drifts) and motion correction parameters were added to the design matrix. Significance maps for each contrast were then overlaid on the flattened cortical surface of each individual hemisphere. On these flat maps, the borders between dorsal and ventral parts were set at the midline of the representation of the horizontal meridian coinciding with the calcarine sulcus, the borders between V1/V2 and V2/V3 were set at the midline of the representations of the vertical and horizontal meridian, respectively. The obtained ROIs V1d/v, V2d/v, V3d/v of each hemisphere were used for the subsequent ROI analysis. Visual areas V1, V2, and V3 could not be determined in one participant (control subject, C3) due to low levels of BOLD response. Anatomical ROIs for associated visual areas lateral occipital cortex (LOC), fusiform gyrus and inferior temporal gyrus, especially involved in object recognition and face perception (e.g., Grill-Spector et al., 1999; Grill-Spector, 2003; Nagy et al., 2012), were determined by using the automatic parcellation of the cortical surface provided by Freesurfer (aparc.annot; Desikan et al., 2006). A threshold of 0.5 was applied to determine the minimum fraction of a voxel's volume that must be filled with surface points of a ROI so that the voxel would be included in that ROI. A threshold of 0.5 assures that a voxel cannot belong to more than one ROI. Table 2 shows the mean sizes of the ROIs for both hemispheres for the patient and control group over all sessions. The sizes of the ROIs V2d (*p* = 0.03) und V3v (*p* = 0.007) of the left hemisphere, as well as V1v (*p* = 0.03), V2d (*p* = 0.04) and V3v (*p* = 0.02) of the right hemisphere showed significant differences between patients and controls (see Table 3).

**Table 2:** **Sizes of ROIs, given in mean number of functional voxels (3 × 3 × 3 mm^3^) in the patient group and control group, together with their respective standard errors (SE) for the left and right hemisphere of dorsal (d) and ventral (v) parts of visual areas V1, V2, and V3, as well as for fusiform gyrus, inferotemporal gyrus (ITG) and lateral occipital cortex (LOC)**.

**ROI**	**Left hemisphere**		**Right hemisphere**	
	**Size**	**SE**	**Size**	**SE**
**PATIENTS**				
V1d	40.93	2.07	31.64	2.49
V1v	38.64	3.06	28.78	2.35
V2d	44.51	4.54	32.82	3.09
V2v	31.58	2.41	34.64	3.69
V3d	40.18	3.14	36.67	2.79
V3v	26.11	1.46	25.82	1.54
Fusiform gyrus	96.18	4.26	84.40	3.92
ITG	97.60	3.48	102.53	4.84
LOC	183.78	9.47	173.22	6.35
**CONTROLS**				
V1d	49.67	2.84	51.63	4.15
V1v	45.67	2.14	50.07	3.01
V2d	98.85	8.09	66.33	6.14
V2v	44.56	3.06	47.67	3.40
V3d	72.11	6.21	52.81	1.98
V3v	41.41	1.73	43.48	2.69
Fusiform gyrus	94.94	3.70	86.81	4.60
ITG	106.91	5.73	98.84	2.73
LOC	206.19	7.11	205.12	7.77

**Table 3:** **Results of repeated-measures ANOVAs (significant main effects and interactions) with the three within-subject factors “sessions” (before, during, after training), “kind of stimulation” (flickering checkerboards vs. pictures of everyday objects) and “location of stimulation” (PRL vs. OppPRL) and the between subject factor “group” (patient vs. control) with respect to the dependent variable “percent signal change**.”

	**Main effects**	**Interactions**
**ROI**		
V1	None	Session × location of stimulation [*F*_(4, 44)_ = 4.21; *p* = 0.006]
V2	Session [*F*_(2, 22)_ = 4.34, *p* = 0.026]	Session × location of stimulation [*F*_(4, 44)_ = 3.94; *p* = 0.008]
	Group [*F*_(1, 11)_ = 5.54; *p* = 0.038]	Kind of stimulation × location of stimulation [*F*_(2, 22)_ = 4.98; *p* = 0. 016]
		Location of stimulation × group [*F*_(2, 22)_ = 4.10; *p* = 0.031]
V3	Session [*F*_(2, 22)_ = 4.28, *p* = 0.027]	Session × location of stimulation [*F*_(4, 44)_ = 4.23; *p* = 0.006]
	Group [*F*_(1, 11)_ = 5.46; *p* = 0.039]	Location of stimulation × group [*F*_(2, 22)_ = 4.90; *p* = 0.017]
Fusiform gyrus	Kind of stimulation [*F*_(1, 12)_ = 33.36, *p* < 0.001]	Kind of stimulation × location of stimulation [*F*_(2, 24)_ = 5.12; *p* = 0. 014]
	Group [*F*_(1, 12)_ = 10.35; *p* = 0.007]	Location of stimulation × group [*F*_(2, 24)_ = 3.92; *p* = 0.034]
ITG	Kind of stimulation [*F*_(1, 12)_ = 9.924, *p* = 0.008]	None
LOC	Kind of stimulation [*F*_(1, 12)_ = 10.59, *p* = 0.007]	Session × location of stimulation [*F*_(4, 48)_ = 2.88; *p* = 0.032]
	Location of stimulation [*F*_(2, 24)_ = 9.45, *p* = 0.001]	Location of stimulation × group [*F*_(2, 24)_ = 10.01; *p* = 0.001]

The ANOVAs were conducted separately for visual areas V1, V2 and V3 as well as for fusiform gyrus, inferotemporal gyrus (ITG) and lateral occipital cortex (LOC).

### Voxel-based-morphometry

For data analysis the S(statistical) P(parametric) M(mapping)8 (Wellcome Department of Imaging Neuroscience Group, London, UK; http://www.fil.ion.ucl.ac.uk/spm) software package was used. To investigate longitudinal changes in gray and white matter density due to fixation training we applied the Dartel toolbox (Diffeomorphic Anatomical Registration Through Exponentiated Lie Algebra) implemented in SPM8 (Ashburner, 2007). Before any further analysis the origin of the structural images was set to the anterior-posterior commissure manually. Within every subject its T1-weighted structural scans from the three sessions were spatially realigned to the first image with SPM8 (realignment options were: Quality 0.9, Separation 4, Smoothing (FWHM) 5, Num Pass Register to first and Interpolation 2nd degree B-spline). Then the new segment toolbox in SPM8 was used to generate gray matter-, white matter- and cerebral spinal fluid CSF-images from each one of the realigned structural scans using a very light bias regularization (0.0001) and a bias FWHM cutoff of 60 mm. No cleanup of the resulting images was performed to avoid the accidental remove of important information. The warping regularization parameter was set to 4, the affine registration was made to the ICBM space template - European brains and the sampling distance was set to 4. With the help of the DARTEL-Toolbox from SPM8, in a first step a group-template (patient or control, respectively) and corresponding flow-fields for every subject were calculated from the segmented images within the group control and patient. We decided to generate different separate templates for each group to avoid that group-specific differences are diminished during this process. The parameters for this setting were as follows: Regularization form: linear elastic energy, six outer iterations with the following respective settings for inner iterations, registration parameters, time steps and smoothing parameter: (3, [4, 2, 1e-6], 1, 16), (3, [2, 1, 1e-6], 1, 8), (3, [1, 0.5, 1e-6], 2, 4), (3, [0.5, 0.25, 1e-6], 4, 2), (3, [0.25, 0.125, 1e-6],16, 1), (3, [0.25, 0.125, 1e-6], 64, 0.5). The optimization settings were: 0.01 for the LM regularization, 3 cycles, and 3 iterations. The segmented images of each subject were then normalized to the MNI space based on the flow-fields and the corresponding group template that were generated in the first DARTEL step. This was done with the “Normalize to NMI Space” module of the DARTEL toolbox. The spatially normalized images were then modulated with the Jacobi determinants of the deformations to preserve the signal amount in the images. Finally, the resulting images were smoothed with an 8-mm FWHM Gaussian kernel.

For statistical analysis two general linear models were constructed, for white and gray matter, respectively. Using the factorial design specification batch in SPM8, the “full factorial” design was selected in both cases, with the only factor being controls vs. patients. We assumed independence and unequal variance for both model specifications. Level one and level two in the design were the smoothed, normalized gray, and white matter likelihood maps for the controls and the patients, respectively, with each of the three sessions in ascending order. In an additional covariate we tried to capture the training effect by setting their values to “1”, “2”, and “3” for the first, second, and third sessions, while applying an overall mean centering for all subjects. Finally, for explicit masking we used the brainmask.nii image provided by SPM8. To assess group effects or differences one and two sample *t*-tests were conducted. Only clusters surviving a statistical threshold of less than 0.0001 (uncorrected for multiple comparisons) on voxel level and less than 0.05 [(F)false (D)discovery (R)rate corrected] on cluster level are supposed to be significant and are reported. Significant clusters are visualized on a volume-based standard brain from a single normal subject (ch2.nii.gz), using the software MRIcron (Rorden and Brett, 2000; http://www.nitrc.org/projects/mricron).

## Results

### Fixation stability

**Figure 3:**
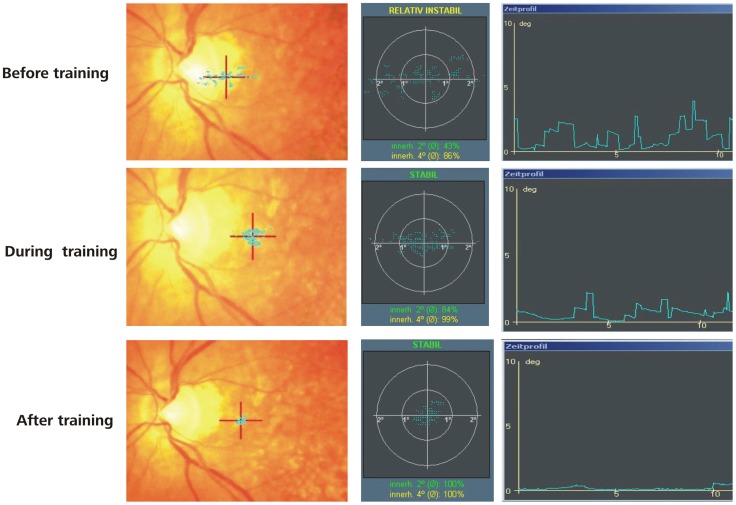
**Results of microperimetry and fixation stability measurements**. An example is given for the development of fixation stability over training in a typical AMD Patient. The PRL is located at the red cross. The blue dots represent individual fixations and their spatial variability reflects fixation stability. An improvement in fixation stability can be observed over the training period in this patient.

**Figure 4:**
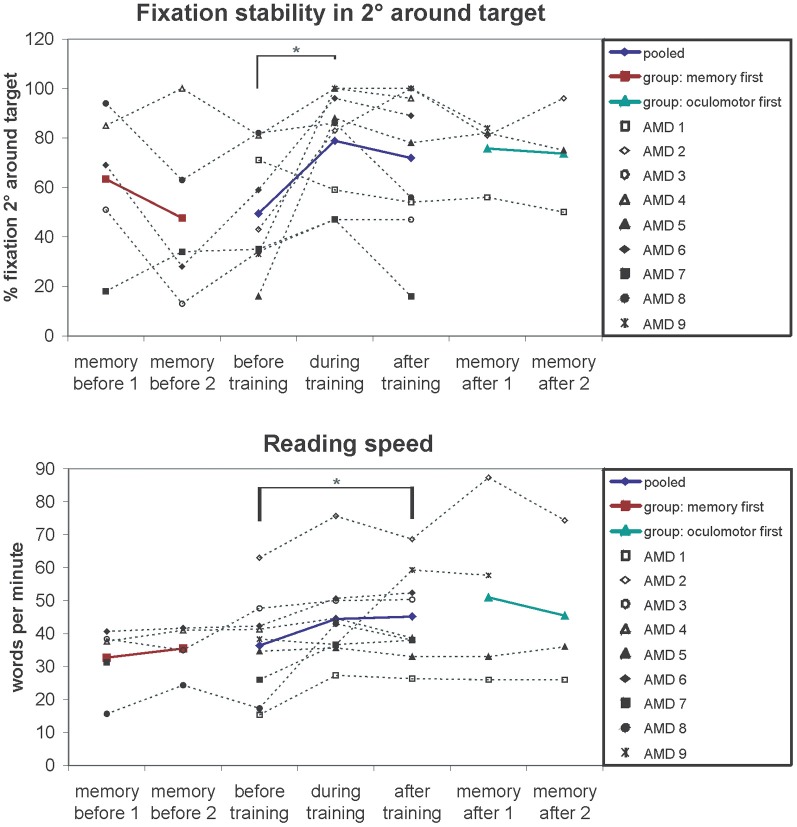
**Fixation stability (% fixation around 2 degrees of target) and reading speedfor the successive measurements periods depicted for individual patients (symbols, connected by dotted lines) and group means (colored lines)**. The results show improved fixation stability after training (significant main effect of session *p* = 0.01). Pairwise comparisons show a significant increase in fixation stability after on average 3 months of training (*p* = 0.04). The memory training has no effect on fixation stability regardless of whether memory training was performed before (memory first *p* = 0.29) or after fixation training (memory after *p* = 0.93), ruling out non-specific placebo effects. A similar pattern can be observed for the reading speed. Reading speed increases over the 6 months of training (significant main effect of session *p* = 0.02). Auditory memory training had no effect on reading speed regardless of whether memory training was performed before (memory first *p* = 0.41) or after fixation training (memory after *p* = 0.57).

Changes in fixation stability were assessed with the Nidek MP-1 microperimeter (Nidek Co, Japan) as described in the methods section. Figure 3 shows an example of the development of fixation stability in one patient (AMD2) from before training, after 3 months training and after 6 months training. The left and middle columns show the distribution of fixation samples around the target, with the middle column additionally giving percentages of fixation samples falling in a range of 2° or 4° diameter visual angle around the target. We used the percentages of fixation points that fell in a range of 2° diameter visual angle around the center of the target as measure for fixation stability, in this patient (AMD2) increasing from 43% before training to 100% after training. The right column in Figure 3 gives the respective time profile of the first 10 s of fixation, which also improved substantially for patient AMD2 over the training period of 6 months. Figure 4 gives an overview of the mean development in fixation stability for all nine AMD patients. To assess immediate training effects of the eccentric viewing training, fixation stability data in Figure 4 was pooled across the group that started with the auditory memory training and the group that started with the eccentric viewing training for the three sessions immediately “before training,” “during training,” and “after training” (*n* = 9), with the session “during training” and “after training” on average 3 months or 6 months after the start of the training, respectively. AMD5 had an additional measurement of fixation stability 7 weeks after the start of the oculomotor training (fixation stability of 82% within 2° degrees around target), not shown in Figure 4. The two remaining sessions, in which both groups completed the auditory memory training, were analysed separately for the group that started with the auditory memory training (sessions “memory before 1” and “memory before 2”; *n* = 5) and for the group who did the auditory memory training after the eccentric viewing training (sessions “memory after 1” and “memory after 2”; *n* = 4, patient AMD9 has, at the time of submission, not yet completed the last session “memory after 2”).

Fixation stability of normally sighted controls was measured once yielding to values between 97 and 100%.

We conducted a repeated-measures ANOVA with the within-subject factor “session” (before, during, after) and the between-subject factor “training group” (oculomotor first, memory first). The analysis yielded a significant main effect “session” [*F*_(2, 14)_ = 6.54; *p* = 0.01]. Pair-wise comparisons showed a significant difference between the session “before” and “during” training (*p* = 0.04). The main effect “training group” and the interaction “session” × “training group” were not significant (*p* > 0.05).

Both training groups showed no significant differences in fixation stability between the two sessions conducted during auditory memory training (all *p* > 0.05).

### Reading speed

For measuring reading speed patients read aloud a continuous text for 3 min, which was recorded. We then counted the number of words read and calculated the mean number of words read per minute, as described in the methods section. Both patients (1.05/min) and controls (0.48/min) did not make more than one reading error per minute on average, leading us to exclude reading error rates in further analyses. Controls completed only one reading test (mean reading speed 127 words per minute; *SE* = 4.99). Mean values of reading speed for the patients were calculated in the same way as described for fixation stability over five sessions (AMD9 has not yet completed session 5) and depicted in Figure 4. We conducted a repeated-measures ANOVA with the within-subject factor “session” (before, during, after) and the between-subject factor “training group” (oculomotor first, memory first). The analysis yielded a significant main effect “session” [*F*_(2, 14)_ = 5.2; *p* = 0.020]. The main effect “training group” and the interaction “session” × “training group” were not significant (*p* > 0.05).

Both training groups showed no significant differences in reading speed between the two sessions conducted during auditory memory training (all *p* > 0.05).

### Visual acuity

**Figure 5:**
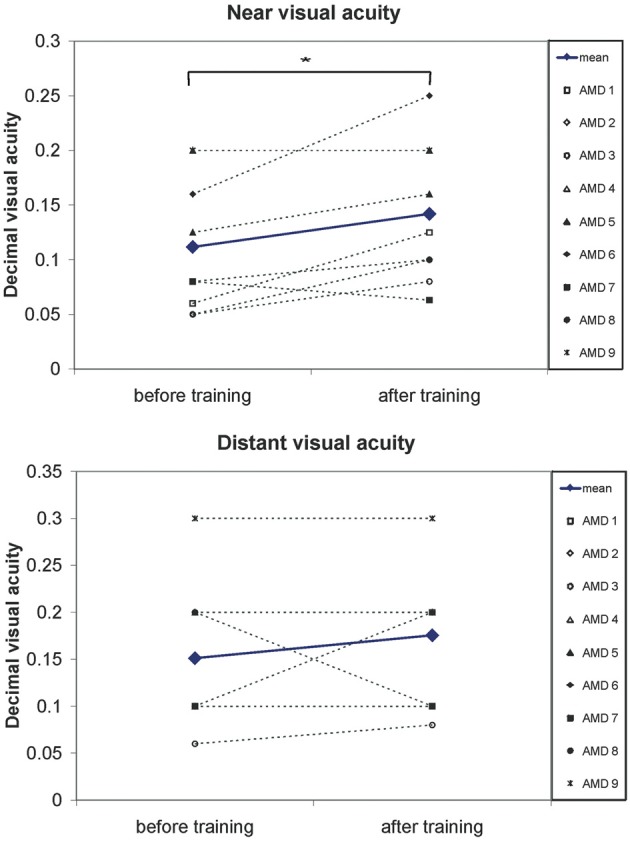
**A significant increase in visual acuity after the 6 months of training was observed**. Pairwise comparisons show a significant increase in near visual acuity (*p* = 0.03) but not for distant visual acuity (*p* = 0.3). Symbols, connected with dotted lines, show the values of individual patients, blue lines show the mean values over all patients.

Near and distant visual acuity were assessed at two time points—before the start and after completion of eccentric viewing training, as described in the methods section. Figure 5 shows the mean development of visual acuity over those two time points for all nine AMD patients. A significant increase can be seen in near visual acuity [*t*_(8)_ = 2.66; *p* = 0.029].

### Visual function questionnaire

**Figure 6:**
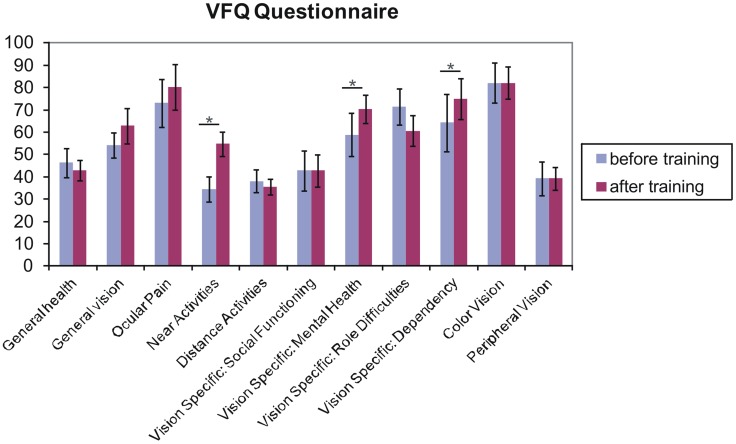
**Results from the Visual Functioning Questionnaire**. One sided *t*-test revealed that fixation training significantly improved subjects' judgments of their near visual activities, their vision-specific mental health (depressive symptoms due to the vision deficit) and their vision specific dependency (e.g., rely on the help of others because of vision deficit) (*p* < 0.05). Other scales do not differ over training. Error bars reflect standard errors.

Figure 6 shows the obtained mean values of the subscales of the Visual Function Questionnaire (VFQ-25; Mangione et al., 2001), as collected from seven patients (for AMD1 and AMD2 did not complete the questionnaires). Three subscales (near activities, *p* = 0.014; vision specific: mental health, *p* = 0.047; vision specific: dependency, *p* = 0.046) showed significant improvements in one-sided *t*-tests.

### fMRI analysis

#### BOLD signal in V1, V2, and V3 while directly stimulating PRL and OppPRL

**Figure 7:**
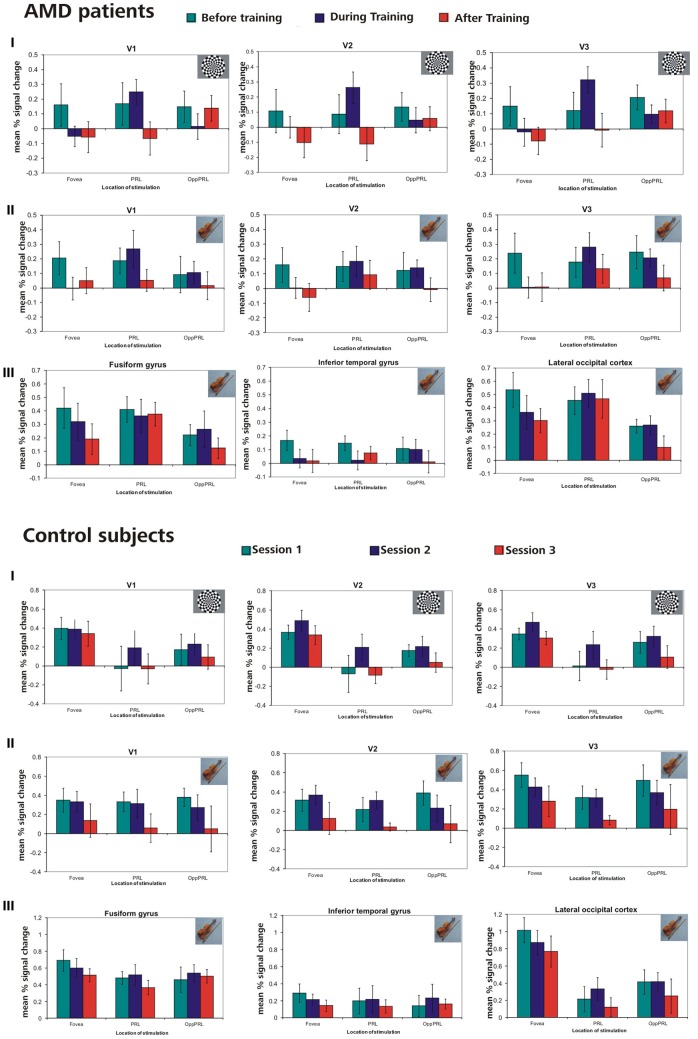
**Mean % signal changes (vs. baseline) in AMD patients and normally sighted controls before, during, and after fixation training in the individual primary (V1), secondary (V2), and tertiary (V3) visual cortex and in the anatomically predefined higher visual areas in the inferior temporal gyrus (ITG), the lateral occipital cortex (LOC), and the fusiform gyrus (as labeled in Freesurfer)**. The stimuli were either checkerboards (I) or meaningful pictures (II, III). For checkerboards % signal change is only reported for the individual primary cortices because no significant activation was found in higher visual areas for that kind of stimulus. The stimulus either appeared in the fovea, the PRL or the OppPRL. In the control subjects, sessions 1, 2, and 3 refer to the corresponding sessions of the individually matched AMD patients. Thus, if the corresponding AMD patient started with the oculomotor training, sessions 1, 2, and 3 refer to the first, second and third fMRI session for the matching control subject. If the corresponding AMD patient had the oculomotor training after the sham training, sessions 1, 2, and 3 in the figure refer to the third, fourth and fifth fMRI session for that control.

In this analysis we were interested in the BOLD responses when the PRL and a comparably peripheral area in the opposite hemifield, the OppPRL, were directly stimulated. We compared activation in the respective projection zones of the PRL and the OppPRL in the visual cortex, namely visual areas V1, V2, and V3. We also compare these activations to those evoked by stimulation in the fovea (scotomatous region in patients). Accordingly, for the group of patients with the PRL in the lower visual field and their respective controls, we considered BOLD activation in the dorsal portions only, pooled over both hemispheres, as the PRL projection zone, and ventral portions only as the OppPRL projection zone, also pooled over hemispheres. For the patients with the PRL in the left visual field and their respective controls, we considered BOLD activation in the right hemisphere only as the PRL projection zone and BOLD activation in the left hemisphere only as the OppPRL projection zone, pooled over dorsal and ventral portions. For the patient with her PRL in the right visual field and her control, we considered BOLD activation in the left hemisphere only for the PRL projection zone and in the right hemisphere only for the OppPRL projection zone, again pooled over dorsal and ventral portions. We computed % signal change of the BOLD signal for each condition (stimulation with flickering checkerboards or pictures of everyday objects at the PRL or OppPRL) and session, each as the difference to baseline. Figure 7 shows the mean % signal change values as a difference to baseline for all conditions and the sessions “before,” “during” and “after training,” for the patient and control group, respectively. The data from one patient (AMD 7) was excluded from this analysis, because she showed strongly negative BOLD responses.

We computed repeated-measures ANOVA with the three within-subject factors “sessions” (before, during, after training), “kind of stimulation” (flickering checkerboards vs. pictures of everyday objects) and “location of stimulation” (Fovea, PRL, OppPRL) and the between subject factor “group” (patients vs. normal-sighted controls) with respect to the dependent variable “percent signal change”, separately for visual areas V1, V2, and V3. The results are given in Table 3.

To test for possible changes in BOLD signal during sham training, we conducted a repeated-measures ANOVA with the within subjects factors visual area (V1, V2, V3), session (memory 1, memory 2), kind of stimulation (checkerboards, pictures) and location of stimulation (fovea, PRL, OppPRL), for both training groups (oculomotor first or memory first) separately. No main effects or interactions reached statistical significance (all *p* > 0.05).

**Figure 8:**
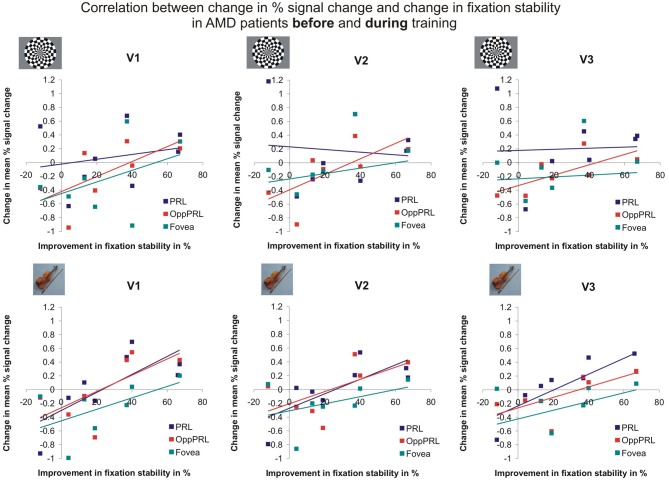
**Correlation between the change in % signal change and change in fixation stability, for the patient group only, before fixation training and during fixation training in individually mapped primary (V1), secondary (V2), and tertiary (V3) visual cortex**. The upper row depicts the correlation between changes in % signal change and changes in fixation stability in case of checkerboard stimulation, the lower row depicts these correlations for everyday images. The differently colored symbols refer to the different retinal locations that were stimulated (green, fovea; red, OppPRL; blue, PRL).

**Figure 9:**
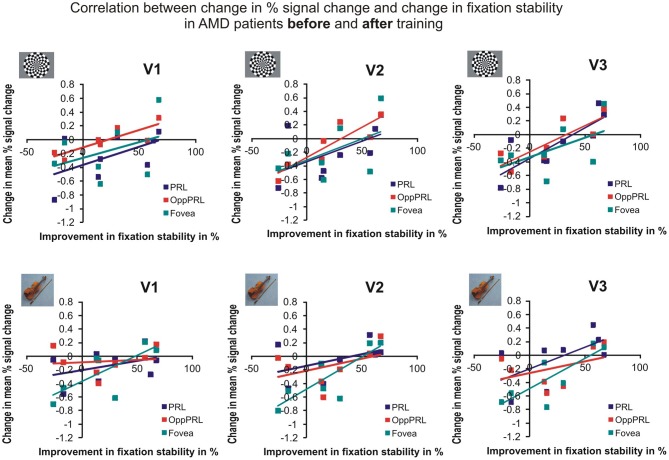
**Correlation between the change in % signal change and change in fixation stability for the patient group only before fixation training and after fixation training in individually mapped primary (V1), secondary (V2), and tertiary (V3) visual cortex**. The upper row depicts the correlation between changes in % signal change and changes in fixation stability in case of checkerboard stimulation, the lower row depicts these correlations for everyday images. The differently colored symbols refer to the different retinal locations that were stimulated (green, fovea; red, OppPRL; blue, PRL).

To examine the relation between BOLD signal and training effects, we correlated the increase in fixation stability between the sessions “before training” and “during training,” as well as between the sessions “before training” and “after training,” with the individual differences in % signal change in the BOLD signal between the same two sessions, respectively. The results for the correlations between sessions “before training” and “during training” can be seen in Figure 8. Improvements in fixation stability correlate significantly with increases in BOLD response, but only, when the PRL is stimulated with meaningful object pictures (see Table 4, upper left panel). The results for the correlations between sessions “before training” and “after training” can be seen in Figure 9. Significant correlations between improvements in fixation stability and increases in BOLD response are marked in bold font (see Table 4, lower left panel). A consistent significant positive correlation can be seen for the stimulation of the OppPRL area with flickering checkerboards, a trend that can already be observed for the comparison between the sessions “before” and “during training,” but that does not reach statistical significance. The data from patient AMD 7 were also excluded from this analysis, because she showed strongly negative BOLD responses.

**Table 4:** **Pearson correlation coefficients and respective *p*-values (in brackets) for the correlation between difference in % signal change and difference in fixation stability (A) between the sessions “before training” and “during training” and (B) between the sessions “before training” and “after training**.”

	**Flickering checkerboards**			**Pictures**				**Flickering checkerboards**			**Pictures**		
	**Fovea (*N* = 7)**	**PRL (*N* = 8)**	**OppPRL (*N* = 7)**	**Fovea (*N* = 7)**	**PRL (*N* = 8)**	**OppPRL (*N* = 7)**		**Fovea (*N* = 7)**	**PRL (*N* = 8)**	**OppPRL (*N* = 7)**	**Fovea (*N* = 7)**	**PRL (*N* = 8)**	**OppPRL (*N* = 7)**
**(A) Correlations for differences between sessions “before training” and “during training.”**													
***V1***	0.416 (0.353)	0.220 (0.601)	0.645 (0.118)	0.541 (0.210)	0.**745** (0.034)	0.666 (0.102)	***Fusiform gyrus***	0.234 (0.614)	−0.087 (0.837)	0.148 (0.751)	0.470 (0.288)	0.**766** (0.027)	0.414 (0.356)
***V2***	0.194 (0.677)	−0.099 (0.815)	0.691 (0.085)	0.401 (0.373)	0.**753** (0.031)	0.588 (0.165)	***ITG***	−0.110 (0.815)	−0.433 (0.284)	−0.106 (0.821)	0.520 (0.231)	0.705 (0.051)	0.056 (0.905)
***V3***	0.068 (0.886)	0.045 (0.916)	0.711 (0.073)	0.406 (0.366)	0.**840** (0.009)	0.66 (0.101)	***LOC***	0.292 (0.525)	−0.131 (0.758)	0.659 (0.108)	0.458 (.302)	0.**859** (0.006)	0.642 (0.120)
**(B) Correlations for differences between sessions “before training” and “after training.”**													
***V1***	0.376 (0.406)	0.54 (0.166)	0.**838** (0.019)	0.735 (0.060)	0.391 (0.339)	0.134 (0.775)	***Fusiform gyrus***	0.638 (0.123)	0.547 (0.161)	0.751 (0.052)	0.**859** (0.013)	0.536 (0.171)	0.**777** (0.040)
***V2***	0.509 (0.244)	0.545 (0.163)	0.**885** (0.008)	0.**827** (0.022)	0.449 (0.265)	0.428 (0.338)	***ITG***	0.495 (0.258)	0.400 (0.326)	0.610 (0.146)	0.685 (0.089)	0.445 (0.269)	0.092 (0.844)
***V3***	0.533 (.218)	0.**813** (0.014)	0.**825** (0.022)	0.**810** (0.027)	0.586 (0.127)	0.410 (0.360)	***LOC***	0.266 (0.564)	0.397 (.331)	0.**775** (0.041)	0.748 (0.053)	0.070 (0.869)	−0.031 (0.948)

Left columns: correlation coefficients for visual areas V1, V2, and V3. Right columns: correlation coefficients for anatomical areas fusiform gyrus, inferior temporal gyrus (ITG) and lateral occipital cortex (LOC). Statistically significant correlations are marked in bold font.

#### Bold-responses in anatomical areas fusiform gyrus, inferior temporal gyrus and LOC

We examined activation in the respective projection zones of the fovea, the PRL, and the OppPRL in anatomical areas fusiform gyrus, inferior temporal gyrus (ITG) and LOC. Thus, for the patients with the PRL in the left visual field and their respective controls, we considered BOLD activation in the right hemisphere only as the PRL projection zone and BOLD activation in the left hemisphere only as the OppPRL projection zone. For the patient with her PRL in the right visual field and her control, we considered BOLD activation in the left hemisphere only for the PRL projection zone and in the right hemisphere only for the OppPRL projection zone. For the group of patients with the PRL in the lower visual field and their respective controls, we pooled over both hemispheres for both, the PRL projection zone and the OppPRL projection zone. We computed % signal change of the BOLD signal for each condition (stimulation with flickering checkerboards or pictures of everyday objects at the fovea, the PRL or OppPRL) and session, each with respect to baseline.

We computed repeated-measures ANOVA with the three within-subject factors “sessions” (before, during, after training), “kind of stimulation” (flickering checkerboards vs. meaningful pictures) and “location of stimulation” (Fovea, PRL, OppPRL) and the between-subject factor “group” (patients vs. normal-sighted controls) with respect to the dependent variable “percent signal change,” separately for areas fusiform gyrus, ITG and LOC. The results are given in Table 3, lower panel. For all three areas fusiform gyrus, ITG and LOC pictures of everyday objects yielded, as expected, significantly more BOLD response than flickering checkerboards. Figure 7 shows the mean % signal change values as a difference to baseline for the stimulation with everyday pictures and the sessions “before,” “during” and “after training,” for the patient and control group, respectively. The data from one patient (AMD 7) were again excluded due to strongly negative BOLD responses.

To test for possible changes in BOLD signal during sham training, we conducted a repeated-measures ANOVA with the within subjects factors anatomical area (fusiform gyrus, ITG, LOC), session (memory 1, memory 2), kind of stimulation (checkerboards, pictures) and location of stimulation (fovea, PRL, OppPRL), for both training groups (oculomotor first or memory first) separately. Both training groups showed a significant main effect “anatomical area” (*p* = 0.014 and *p* = 0.001, respectively). The main effect “session” was not significant (*p* = 0.178 and *p* = 0.761). Only the training group that conducted the oculomotor training first showed an additional significant main effect “kind of stimulation” (*p* = 0.014), and a significant interaction “anatomical area × kind of stimulation” (*p* = 0.009).

**Figure 10:**
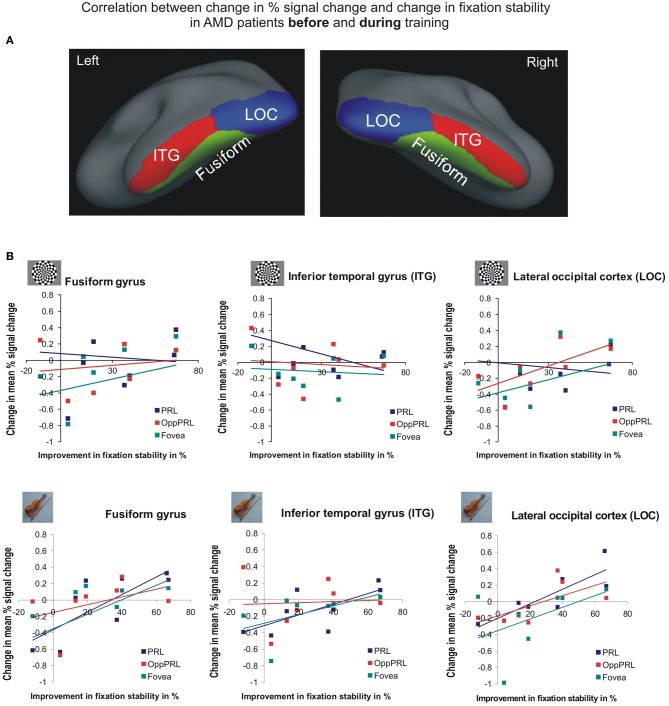
**Correlation between the change in% signal change and change in fixation stability for the patient group only before fixation training and during fixation training in the inferior temporal gyrus (ITG), the lateral occipital cortex (LOC), and the fusiform gyrus [(A) as labeled in Freesurfer]. (B)** The upper row depicts the correlation between changes in % signal change and changes in fixation stability in case of checkerboard stimulation, the lower row depicts these correlations for everyday images. As in Figures 8, 9, the differently colored symbols refer to the different retinal locations that were stimulated (green, fovea; red, OppPRL; blue, PRL).

**Figure 11:**
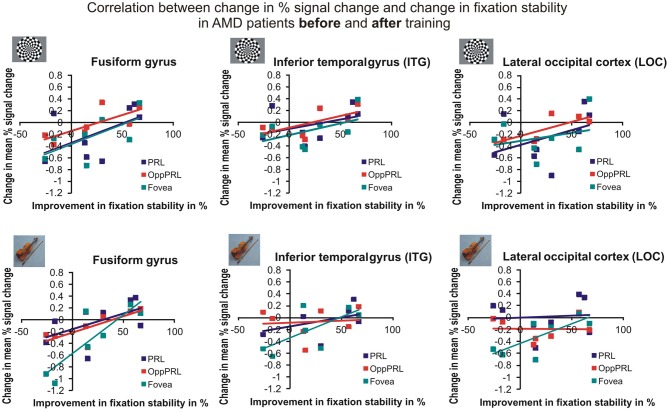
**Correlation between the change in % signal change and change in fixation stability for the patient group only before fixation training and after fixation training in the inferior temporal gyrus (ITG), the lateral occipital cortex (LOC), and the fusiform gyrus**. The upper row depicts the correlation between changes in % signal change and changes in fixation stability in case of checkerboard stimulation, the lower row depicts these correlations for everyday images. As in Figures 8, 9, the differently colored symbols refer to the different retinal locations that were stimulated (green, fovea; red, OppPRL; blue, PRL).

To investigate the relationship between BOLD signal and training effects we correlated the increase in fixation stability between the sessions “before training” and “during training,” as well as between the sessions “before training” and “after training,” with differences in % signal change between the two sessions, respectively. The results can be seen in correlation coefficients (Figures 10, 11, and Table 4, upper and lower right panel). Also in areas fusiform gyrus and LOC improvements in fixation stability between the sessions “before” and “during training” correlate significantly with increases in BOLD response when the PRL is stimulated with pictures of everyday objects. For the correlations between the sessions “before” and “after training” there is no systematic trend observable. The data from patient AMD 7 were also excluded from this analysis, because she showed strongly negative BOLD responses.

### Voxel-based morphometry results

**Figure 12:**
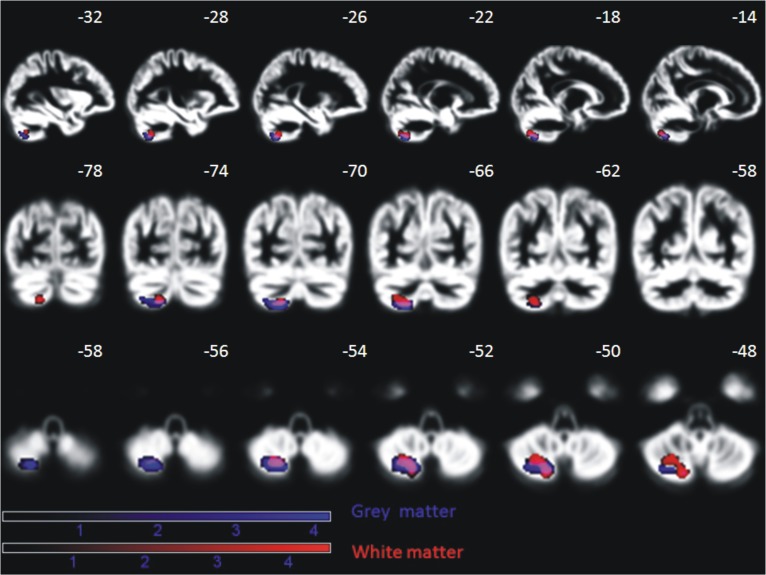
**Significant increase of gray and white matter in the posterior cerebellum of nine AMD patients over the period of oculomotor training for, on average, 6 months (*p* < 0.05, FDR corrected on cluster level)**. This effect is absent in age-matched controls. Effects are depicted on a mean normalized gray matter image of all subjects (patients and controls). The MNI coordinates in terms of millimeter deviation from the anterior commissure are presented.

A positive effect of training was reflected in brain increase in gray and white matter density in the left posterior cerebellum (semi-lunar lobule, Crus II) for the patient group (gray matter: peak MNI coordinate (*x* = −20, *y* = −67, *z* = −56), cluster size 520 voxel, *Z* = 3.9, p_corr(FDR)_ = 0.034; white matter: peak MNI coordinates (*x* = −20, *Y* = −67, *z* = −53), cluster size 580 voxel, *Z* = 4.2, p_corr(FDR)_ = 0.02) (Figure 12). The control group did not show any sequential effects from measurements 1, 2, and 3 during the same time period, neither for gray nor white matter. A direct group comparison did not yield any significant differences.

## Discussion

The aim of this study was to investigate how a specific training intervention that should lead to a more efficient use of eccentric viewing in patients suffering from AMD could be related to functional and structural brain changes. Behavioral data show a significant improvement in fixation stability, reading speed, and near visual acuity as a result of oculomotor training although there is substantial heterogeneity in the amount of benefit individual patients exhibited. Four patients (AMD 2, 5, 6, and 9) showed a clear improvement of fixation stability from before to after the training period. Patients AMD 4 and 8 started already with relatively high stability values and could not increase it much further through the training. Patients AMD 3 and 7 started with low stability values and had room for improvement, but critical life events in these two cases disrupted training, which might be the reason that the patients could not tap their full potential. Patient AMD 1, who did not show improvement in fixation stability over training, preferred to use devices with large magnification at home. He therefore might have lacked sufficient training in eccentric viewing in comparison to the other participants. In contrast reading speed increased in patient AMD 1 as well as in five other patients (AMD 2, 6, 7, 8, and 9) presumably due to training measures. For the three remaining patients (AMD 3, 4, and 5) changes in reading speed appear unsystematic and not directly related to training. Improvements in distant visual acuity were not statistically significant, while improvements in near visual acuity reached statistical significance. Six patients (AMD 1, 2, 3, 5, 6, and 8) showed an increase in near visual acuity, while only 1 patient (AMD 7) showed a slight decrease and two patients (AMD 4 and 9) maintained the same near visual acuity values before and after the training. The patients as a whole reported that they benefited from the training with respect to their near visual activities, as demonstrated by their responses in the VFQ-25 questionnaire (Mangione et al., 2001). But one must note that during training some of the patients started to use a hyperocular for the first time. They subsequently used it during the training, but also in their daily lives. So the significant improvement in the subscale “near visual activities” in the VFQ-25 questionnaire might not be related to the training alone. Our behavioral results are in line with other findings in the literature. In an earlier study (Nilsson et al., 2003) AMD patients with large absolute scotomata were trained in a reading task to establish a more favorable retinal locus on their eccentric retina for reading. As a consequence of training patients' reading speed increased. Nguyen et al. (2011) reported a study where the fixation behavior of patients with juvenile forms of macular degeneration was randomly trained with either a rapid serial visual presentation task or a low vision sensorimotor reading task. Both tasks led to an improvement in fixation behavior. Usually in studies on rehabilitation methods for patients with central vision loss it could be shown that an improvement in reading speed was accompanied by a stabilization of eccentric fixation (e.g., Sunness et al., 1996; Trauzettel-Klosinski and Tornow, 1996; Nilsson et al., 1998; Crossland et al., 2004; Rubin and Feely, 2009). Although in our study we also observed a significant increase in both—reading speed and fixation stability—across all patients due to training, surprisingly the amount of individual improvements in fixation stability was not directly correlated to individual improvements in reading speed (*r* = −0.592; *p* = 0.12). It is unclear at the moment what the reason for this could be. With regard to improvements in visual acuity, another study of Polat (2009) could also show that patients with amblyopia could benefit from a visual perceptual learning task with respect to their visual acuity and contrast sensitivity. Ishiko et al. (2010) showed an improvement in visual acuity for the worse eye in patients with age-related maculopathy over time, without having applied any training methods. While the better eye was deteriorating, this effect could in part be attributed to a more stable eccentric fixation with the worse eye. Overall, it could be shown that at least a subgroup of the AMD patients in our study showed substantial improvements in behavioral parameters over the training period. But the results also hint to the difficulty of the patients to hold on to their achieved standards without further active oculomotor training, as we observed some decline in measurements during the sham training after the oculomotor training phase.

Fixation stability also showed a significant positive correlation with brain activation changes in the individual primary, secondary, and tertiary visual cortex, the fusiform gyrus and the lateral occipital lobe during the initial phase of the training, where the increases in fixation stability were most pronounced (comparison of sessions “before” and “during training”). Interestingly this PRL-specific positive correlation was only present when the PRL was stimulated with semantically meaningful pictures but not with checkerboard stimuli. Earlier findings suggest that active tasks lead to better stimulus-correlated activation in the PRL projection zone compared to the results for passive tasks (e.g., Masuda et al., 2008; Liu et al., 2010; Plank et al., 2012). Even if the task in this study only demanded passive viewing, the semantic quality of the stimuli seems to play an essential role with respect to the signal in the PRL projection zone. One reason could be that these stimuli have a more relevant character in patients' daily lives than abstract radial checkerboard stimuli and thus the former activate more top-down processing yielding greater BOLD responses. The effect could partially also be due to enhanced attention as oculomotor training may assist the patients to guide their attention to visual input at the PRL. Overall, our data show that the patients who showed the most pronounced increase in fixation stability were also the ones who showed an increase in BOLD signal for measurements made “before” and “during training,” while the patients who showed no or only a slight improvement in fixation stability showed no increase or even a decrease in BOLD signal, as depicted in Figures 8, 10. This positive correlation between change in BOLD signal and improvement in fixation stability is also evident in the comparison between the sessions “before training” and “after training” (see Figures 9, 11), even though the correlations for several visual areas and conditions now lack statistical significance (see Table 4). It can be seen that especially the PRL-specific increase in BOLD signal that corresponded with the most pronounced increase in fixation stability between the two time points “before” and “during training” does not present itself as a sustainable effect up to the time point “after training.” We assume that a general tendency of the BOLD response to decline over time, as discussed below with respect to the effects of sequential testing, might superimpose onto possible training effects to a certain extent. This could be attributed to habituation or attention effects related to doing the same task several times. Also the fact that we used the same set of pictures in our stimulation at each of the sessions could play a role here. Inspection of Figure 7 reveals that repeated measurements in the controls also led to lower BOLD responses over time. These changes occurred in the controls, despite the fact that they did not undergo any training. Thus, the mere repetition of testing has an effect on the BOLD response in visual cortex. Such habituation effects should be taken into account when evaluating the effects of training on cortical responses. On the other hand, Yotsumoto et al. (2008) also found an initial increase in brain activation in the visual cortex of participants in a perceptual learning experiment that was followed by a decline while task performance levels remained high. Indeed, the patients in our study showed the most pronounced increase in fixation stability in the initial phase of the training period, where a PRL-specific correlation with BOLD response increases could be found. Later on most, but not all, patients were able to stabilize their fixation (see Figure 4).

Interestingly and surprisingly, correlation effects between changes in fixation stability and BOLD responses appear to get stronger for non-PRL areas over time (see Table 4). Thus, while we expected a more PRL-specific effect, a more stable eccentric fixation appears to improve the processing of visual information in the entire remaining visual field, not only in the PRL area. Stable fixation could thus help the patients to use their entire visual field more efficiently. In a previous study with patients with central vision loss (Plank et al., 2012) we also found that a group of patients with stable eccentric fixation performed better in a visual search task than another group with unstable eccentric fixation, even across those trials where the target stimuli did not appear in or near their PRL.

The analysis of the Visual Function Questionnaire showed a significant increase in scores with respect to the categories near vision abilities, vision specific mental health (depressive symptoms due to the vision deficit) and vision-specific dependency (e.g., reliance on the help of others because of visual deficit) due to training. Mitchell and Bradley (2006) reported that AMD causes severe decline in life quality. Furthermore, they speculated that the health status and the utility measures referring to the quality of live status in AMD patients are underestimated. Further the authors point out that medical interventions often only reach a small proportion of patients. Another study of Rovner et al. (2009) highlights the need for life quality improvements in AMD patients. In their study AMD patients tended to neglect cognitive, physical and social activities due to their visual deficit, which led to an increased risk of cognitive decline. Indeed in a multi-center study with a large number of JMD and AMD patients, Hernowo et al. (2013) found significant reductions in frontal lobe white matter in patients with AMD (but not patients with JMD), suggesting that AMD could also be associated with cognitive decline.

The results of the present study indicate that life quality could be improved by a suitable oculomotor training not only in terms of visual abilities in general (near vision abilities) but also in terms of general quality of life. Therefore, the training measurement showed a direct impact on at least some aspects of life quality as demanded by Mitchell and Bradley (2006). Such training could lower the risk of cognitive deficits that can be often observed in elderly patients with chronic disease.

We observed increased gray and white matter density in patients with AMD as a consequence of oculomotor training in the left semi-lunar lobule of the cerebellum. This effect was absent in control subjects, which was expected since they did not participate in the oculomotor training. Such a gray matter increase might be related to the eccentric reading training. In a study by Fulbright et al. (1999) the semi-lunar lobule was associated with reading. They showed that the semi-lunar lobule showed increased activation when non-meaningful phoneme assemblies should be read. The area was not active when real words were presented. An alternative explanation could be that the executive control of eye movement was enhanced due to training. Habas et al. (2009) showed in a model-free approach that the semi lunar lobule is part of the executive control network during resting state.

An fMRI study with AMD patients indicated that a reading task led to increased activation in the prefrontal and the parietal cortex (Szlyk and Little, 2009). Another study of Little et al. (2008) revealed that AMD patients exhibited decreased activation in the occipital cortex compared to healthy controls and increased activation in the prefrontal cortex and the intraparietal sulcus while they were executing saccades or pursuit eye movements. During pursuit eye movement there was also additional increased activation in the frontal and supplementary eye fields. In both studies executive tasks were performed but no cerebellar activations were reported. It is, however, well established that the cerebellum plays an essential role in eye movements and executive control. The increased gray matter density in the inferior cerebellum might reflect a general effect of learning rather than a specific one, since the semi-lunar lobule is not directly linked to eye movements *per se*.

### Effects of sequential scanning over a 1-year period

Overall 7 control subjects were recruited, who were matched on age and gender to the respective patients. Although they did not participate in the oculomotor or eccentric reading training they participated in the same fMRI measurements over the same period of time. We wanted to rule out any sequential effects that could take place by repeating the same measurements five times over a 12-month period. The results of the controls, together with the results of the patient group, are presented in Figure 7 and indicate that indeed there are some sequential effects of repeated testing. As Table 3 shows, there are significant main effects of the factor session for areas V2 and V3, as well as significant interactions between the factors session and location of stimulation, in regard to whether the fovea, the PRL or the OppPRL were stimulated, in areas V1, V2, V3, and LOC. The BOLD response exhibits a tendency to decline over time, suggesting a form of habituation, which is most pronounced when the fovea is stimulated. There is a slight tendency that the BOLD response due to stimulating the PRL is enhanced in the second session, which might be caused by attention effects or perceptual learning in the periphery (Yotsumoto et al., 2008). With the exception of the LOC no ROI shows a significant main effect of location of stimulation. Areas V2, V3 and fusiform gyrus show a significant main effect for group and areas V2, V3, fusiform gyrus and LOC show a significant interaction of location of stimulation and group, which points to an overall reduced neural response in the patients in these areas when the fovea is stimulated. Nevertheless, the patients showed some positive signal even when the stimuli fell in an area covered by their scotoma. An explanation could be that the scotomata are not symmetrical in all cases and some residual vision could be spared at the rim of the scotomata, leading to some residual perception in the patients when this area is stimulated with high-contrast stimuli as were used here. Part of the significant interaction between location of stimulation and group can also be attributed to the difference between PRL and OppPRL stimulation that only the patient group shows, since the significant interaction also holds when the fovea is excluded from the analysis in the fusiform gyrus (*p* = 0.049) and in the LOC (*p* = 0.028). The difference between groups (patients vs. controls) is also no longer significant, when the BOLD responses of the foveal stimulation are excluded (all *p* > 0.05). Together with the clear correlation effects between individual improvements in fixation stability and increase in BOLD signal over the same period of time, especially during the initial phase of the training, we therefore conclude that the training effects reported in the patient group cannot be accounted for by mere repetition effects.

## Summary

In this study we trained AMD patients to improve their fixation stability using specific oculomotor training protocols. Patients benefited from training with respect to their fixation stability, reading speed and near visual acuity. Training also led to an improvement in some aspects of the patients' quality of daily life. After training, patients reported that they needed less help from others and were more autonomous than before training. Training led to significant albeit modest changes in brain functions and structure. We found a positive correlation between fixation stability and an increase in neural response in the PRL projection zones in AMD patients during the initial phase of the training. Interestingly structural changes were also found in the cerebellum of these patients following oculomotor training. Increases of gray and white matter density in the cerebellum were found that might be due to learning to perform visual tasks with eccentric viewing. Overall the study showed that patients with AMD can benefit from specific oculomotor training procedures. Such training is associated with measureable changes in brain functions and structure.

### Conflict of interest statement

The authors declare that the research was conducted in the absence of any commercial or financial relationships that could be construed as a potential conflict of interest.
